# Effects of cyclotide-producing plants on the development and life history parameters of *Leipothrix violarius*

**DOI:** 10.1007/s10493-025-01075-x

**Published:** 2025-10-22

**Authors:** Anna Pińkowska, Patrycja Jankiewicz, Katarzyna Wójcik, Elżbieta Wójcik-Gront, Mariusz Lewandowski

**Affiliations:** 1 Department of Plant Protection, Warsaw University of Life Sciences, Nowoursynowska 159, Warsaw, 02-776 Poland; 2 Department of Biometry, Warsaw University of Life Sciences, Nowoursynowska 159, Warsaw, 02-776 Poland

**Keywords:** Eriophyoid mites, Host range, Life-table parameters, Model organism, Violaceae

## Abstract

*Leipothrix violarius* (Acariformes: Eriophyoidea) is a phytophagous mite specialized in feeding on plants from the genus *Viola*. It has been recorded on five host species, including *Viola uliginosa*, which is rare or endangered in much of its range. Despite taxonomic revisions, little is known about the biology or host specificity of *L. violarius*. In this study, we compared the effects of *V. uliginosa* and *V. odorata* on the development, survival, and population parameters of *L. violarius*, and assessed its host range using selected ornamental and naturally occurring violets. Development from egg to adult took approximately seven days on both host plant species, with no sex-related differences. However, mites reared on *V. odorata* exhibited faster egg development and greater population increase per generation than those reared on *V. uliginosa*. Population growth occurred on all tested host species, although mite performance varied. *Viola* plants produce cyclotides—bioactive cyclic peptides with antimicrobial and insecticidal properties. The observed differences in mite performance may reflect interspecific variation in cyclotide content or composition, suggesting a role for these compounds in plant resistance. Due to its narrow host range and epidermal feeding behavior, *L. violarius* offers a promising model for studying plant–mite interactions at the surface level. This system may help clarify how cyclotides mediate and respond to herbivory in *Viola* species.

## Introduction

Understanding the biology of mites, particularly eriophyoid mites (Acariformes: Eriophyoidea), is essential for both fundamental research and practical applications in agriculture and conservation. As obligate plant parasites with high host specificity and often cryptic lifestyles, eriophyoids offer valuable models for studying host–parasite coevolution, plant defence mechanisms, and arthropod adaptation to plant secondary metabolites (Skoracka et al. 2010; De Lillo and Skoracka 2010). From a practical perspective, many eriophyoid species are significant agricultural pests capable of causing extensive crop damage or transmitting plant viruses, often with subtle early symptoms that delay detection (Oldfield and Proeseler 1996; Westphal and Manson 1996; Szydło et al. 2015). Effective management strategies—whether chemical, biological, or based on plant resistance—require detailed knowledge of their life cycles, reproductive strategy, dispersal potential, and host plant interactions (Petanović and Kielkiewicz 2009; Skoracka et al. 2022). Moreover, a growing interest in biodiversity conservation highlights the need to better understand eriophyoids associated with rare or endangered plant species, whose role may range from innocuous cohabitants to overlooked threats (Ozman-Sullivan and Sullivan 2024).

Eriophyoid mites represent one of the most ecologically specialized and economically important groups of plant-feeding mites, second only to spider mites (Tetranychidae) in agricultural relevance (Van Leeuwen et al. 2010). With approximately 5,000 species described to date and new taxa continuously being discovered (Navajas and Ochoa 2013), this superfamily displays remarkable diversity in host specificity, ecological niches, and modes of plant interaction. They predominantly colonize young, metabolically active plant tissues, where their feeding can induce morphological deformations, disrupt physiological processes, and, in severe cases, lead to plant death (Oldfield 1996; Petanović and Kielkiewicz 2009; Westphal and Manson 1996). In addition to direct damage, some species act as vectors of serious plant pathogens (De Lillo and Skoracka 2010; Druciarek and Tzanetakis 2025). The severity of their impact depends not only on mite species but also on host plant genotype and environmental conditions (Rancic, and Petanovic, 2008).

Eriophyoid mites are characterized by an unusually simplified morphology and highly specialized life strategies. Their development is rapid, and most species undergo only two immature stages (larva and nymph) before reaching adulthood, which is atypical among mites (Helle and Wysoki 1996; Lindquist 1996). Developmental time, fecundity, and survival vary depending on host plant species and environmental conditions, particularly temperature and humidity. For example, under optimal laboratory conditions (~ 25 °C), *Aculops lycopersici* (Tryon) completes its development in 6–8 days, with females laying up to 50 eggs during a lifetime of 1–2 weeks (Haque and Kawai 2003). Similarly, *Phyllocoptes adalius* Keifer develops at the same temperature in 7–8 days and shows daily fecundity of around 2 eggs per female (Druciarek et al. 2014). Life-table parameters such as the intrinsic rate of increase (*r*_*m*_) and net reproductive rate (*R₀*) are commonly used to quantify population growth potential, and values of *r*_*m*_ ranging from 0.1 to 0.3 are typical for eriophyoids under favourable conditions (Skoracka et al. 2022). The short generation time and high reproductive rate make these mites effective colonizers but also challenging to manage when they act as pests.

*Leipothrix violarius* (Liro) (syn. *Epitrimerus violarius* Liro), the focus of this study, is a specialist that feeds on species from the *Viola* genus (Liro 1941). Its presence has been recorded on *Viola canina* L., *V. mirabilis* L., *V. montana* L., *V. riviniana* Rchb and *V. uliginosa* Besser in Finland, Hungary, North Russia, and Poland (Farkas 1966; Pińkowska and Lewandowski 2024; Ripka 2007, 2008; Roivainen 1947, 1951). The taxonomic revision of this species was carried out by Pińkowska and Lewandowski (2024) based on the presence of a bifurcate basiventral femoral seta (*bv*) and the results of genomic analysis, which indicated a significant affiliation with the species in the *Leipothrix* genus. A distinctive feature of its feeding is necrosis of epidermal cells, seen as small brown dashes on the leaf surface. As the infestation increases, leaves curl inwards at the edges and eventually dry up. A well-developed population of this mite can lead to progressive plant mortality (Pińkowska and Lewandowski 2024). This is particularly noteworthy as one of its host plants is *V. uliginosa*, which in most countries where it occurs is considered rare or classified as endangered (Baryła and Kuta 2001; Ingelög et al. 1993; Kirschner and Skalnický 1990; Matulevičiūtė 2015; Paul et al. 2016). The occurrence of *L. violarius* on plants from the *Viola* species is intriguing because members of this genus are natural producers of cyclotides, cyclic peptides with potent biological activity and putative roles in plant defense (Craik et al. 1999; Daly et al. 2009; Koehbach and Gruber 2015). These peptides expression patterns vary across species and plant tissues (Slazak et al. 2016). Cyclotides have been detected in the lower and upper epidermal cells (Slazak et al. 2022), where eriophyoid mites feed, yet their influence on those mites’ performance remains unexplored. *L. violarius*, a narrow specialist on violets presents a promising model for investigating how epidermis-localized plant defenses impact arthropod herbivores. To enable such future studies, this work aimed to establish a rearing method and characterize the developmental biology and host range of *L. violarius*.

## Materials and methods

*Viola uliginosa* plants, infested by *L. violarius*, were obtained from the collection of Professor Elżbieta Kuta (Cracow-Ugorek, Poland). These mites were used to establish stock colonies on *Viola odorata *L. and *V. uliginosa*. Before setting up the experiments, specimens from stock colonies reared separately on each plant species were reproduced for three generations in Munger cages, constructed according to Druciarek et al. (2014), allowing synchronized cohorts of females to be obtained. Mite-free plants of *V. uliginosa* were obtained from the same collection, while plants of *V. odorata* were collected on the campus of Warsaw University of Life Sciences (WULS), Warsaw, Poland. Both host plant species were planted in the greenhouse of WULS.

### Developmental time

Experiments on the developmental time and survival of immature stages of *L. violarius* were conducted on detached leaves of *V. uliginosa* and *V. odorata* in Munger cages, following the protocol described by Druciarek et al. (2014). Although we employed standard methods used for rearing herbivorous mites, the laboratory rearing of *L. violarius* and its developmental parameters had not been previously documented. Forty females previously adapted to each host plant species were placed separately in rearing cells containing detached leaves of the respective host plant for 24 h to oviposit. After this period, all females and eggs were removed, leaving one randomly selected egg per cell. The incubation period of eggs, developmental time of juvenile stages, and their survival rate were recorded every 24 h. All adults present at the end of development were slide-mounted in Berlese’s medium, as described by Amrine and Manson (1996). The sex of slide-mounted specimens was determined, and based on the number of females and males, the sex ratio was calculated. Experiments were conducted in a microclimate chamber, Sanyo MLR-350, at a temperature of 25 ± 1 °C, a humidity of 70 ± 10%, and a 16-hour photoperiod (16 h light: 8 h dark).

### Reproductive parameters

Female longevity and reproductive parameters were studied in 60 Munger cages with detached leaves of both host plants (30 for each species). Thirty quiescent female nymphs from the population adapted to *V. odorata* and *V. uliginosa* were transferred separately. Once the nymphs moulted into adult females, one male from separate stock colonies, reared from eggs laid by uninseminated females, was added to each cage. The cages were inspected daily to record the number of laid eggs and the survival of adult mites. Eggs were removed daily, and dead males were replaced with new ones. The deceased specimens were slide-mounted to confirm their sex. It allowed us to decide whether observing specimens in such cages should continue (in the case of male death) or terminate them (if the female had died). This experiment was conducted under the same conditions as above. The number of eggs laid by females and their lifespan determined the total and daily fecundity, female longevity, and pre-, oviposition, and postoviposition periods.

### Population parameters

The data obtained from juvenile developmental time, survival rate, sex ratio, female longevity, and daily fecundity were used to calculate population parameters of *L. violarius* on both plant species. Life tables were constructed from the observed age-specific survival rate (*l*_*x*_) and specific fecundity rate (*m*_*x*_) [net reproductive rate (*R*_*0*_), mean generation time (*T*), doubling time (*TD*), intrinsic rate of population increase (*r*_*m*_), and finite rate of population increase (*λ*)] (Birch 1948; Southwood 1978).

### Range of host plants

Six species of violets were chosen to determine the host range of *L. violarius*. *V. uliginosa* was used as a control plant, along with three ornamental cultivars that were commercially available (*Viola x wittrockiana* Gams ‘Silberbraut’, *V. odorata* ‘Konigin Charlotte’, and *Viola hederacea* Labill), and two commonly occurring in the natural environment habitats of the temperate region, *V. odorata* and *Viola arvensis* Murray, both considered weeds. This selection aimed to (1) include a representative spectrum of natural, ornamental, and phylogenetically diverse *Viola* species, and (2) assess whether *L. violarius* may pose a potential threat to both cultivated and wild growing violets.

For each violet species, 10 plants with at least three well-developed leaves were selected. Ten *L. violarius* specimens (8 females and 2 males) from a stock colony reared on *V. uliginosa* were transferred to one leaf per plant using an eyelash brush. The proportion of females to males was determined based on the result of the sex ratio from the previous experiment. Infested plants were put in a mesh cover to prevent eriophyoid migration and placed in a microclimate chamber Panasonic MLR-352PE, set at 25 ± 1 °C, a humidity of 70 ± 10% and a 16-hour photoperiod (16 h light: 8 h dark). Plants were watered every three days.

After 14 days, all leaves were detached and examined under a stereomicroscope. Mite numbers were recorded, and the population growth rate was calculated based on the formula used by Skoracka et al. (2022): *r* = In(*R*_0_), where *R*_0_ is the finite population growth rate, defined as *n/n*_0_, in which *n*_0_ corresponds to the number of mites placed on each plant at the beginning of the experiment, and *n* corresponds to the number of mites counted after 14 days. If *r* < 0, the population size decreased; *r* > 0, the population size increased, whereas *r* = 0 corresponded to no change in the population size (Skoracka et al. 2022).

### Statistical analysis

Statistical analyses were performed using STATISTICA version 13.0 (TIBCO Software Inc., Palo Alto, California, United States). The Shapiro–Wilk test was used to assess the normality of data distributions. When data did not follow a normal distribution, the non-parametric Mann-Whitney test was used for comparison of two groups. Otherwise, a *t*-test was applied. For comparisons involving more than two groups, the Kruskal-Wallis test was employed, followed by Dunn’s test post hoc for pairwise comparisons. Statistical significance was determined by *p* < 0.05.

To evaluate the variation in population parameters, we applied the bootstrap technique to estimate their distribution through sampling with replacement from the original dataset Klimov and O’Connor (2013). A total of 1000 bootstrap samples were generated by sampling with replacement from the original dataset. For each replicate, the mean and standard deviation (SD) of population parameters (*R*_*0*_, *T*, *TD*, *r*_*m*_, and *λ*) were calculated. One-way ANOVA was performed on these values, followed by Tukey’s test (*α* = 0.05).

In the analysis of the sex ratio comparison, we employed the confidence interval (CI) for a proportion alongside the Z score for two population proportions. Additionally, the Kaplan-Meier method was used to calculate confidence intervals for the estimated survival function at various time points (Kryś and Gront 2021; Sachs et al. 2022). The median number of days for survival was compared between *V. uliginosa* and *V. odorata* through the application of Mood’s median test (Divine et al. 2018).

## Results

### Developmental time and reproductive parameters

Development of *L. violarius*, from egg to adult, did not differ significantly and took around 7 days on both studied host plants (Table 1). However, hatching from eggs occurred significantly faster on *V. odorata*. Survival rate of immature stages was similar for both violet species (Table 2).

**Table 1:** Length of development of *Leipothrix violarius* bred on *Viola odorata* and *Viola uliginosa* (days ± SD)

Development stage	Length of development (days ± SD)				
	*n*	*V. odorata*	*n*	*V. uliginosa*	*p*-value
Egg	45	3.56 ± 0.59	48	4.00 ± 0.77	0.003
Larva	35	1.86 ± 0.49	34	1.74 ± 0.45	0.287
Nymph	32	1.59 ± 0.67	26	1.38 ± 0.57	0.210
Egg-adult	31	6.94 ± 0.89	26	7.35 ± 0.75	0.067
Egg-adult (female)	23	7.00 ± 0.95	21	7.33 ± 0.80	0.217
Egg-adult (male)	8	6.75 ± 0.71	5	7.40 ± 0.55	0.120

**Table 2:** Survival rates of *Leipothrix violarius* bred on *Viola odorata* and *Viola uliginosa*

Development stage	Survival rate (%)					
	*n*	*V. odorata*	*n*	*V. uliginosa*	*p*-value	
Egg	45	0.90 ± 0.04	48	0.96 ± 0.02	0.254	
Larva	35	0.78 ± 0.06	34	0.71 ± 0.07	0.505	
Nymph	31	0.89 ± 0.05	26	0.76 ± 0.08	0.192	
Egg-adult	31	0.62 ± 0.08	26	0.52 ± 0.11	0.447	

**Fig. 1:**
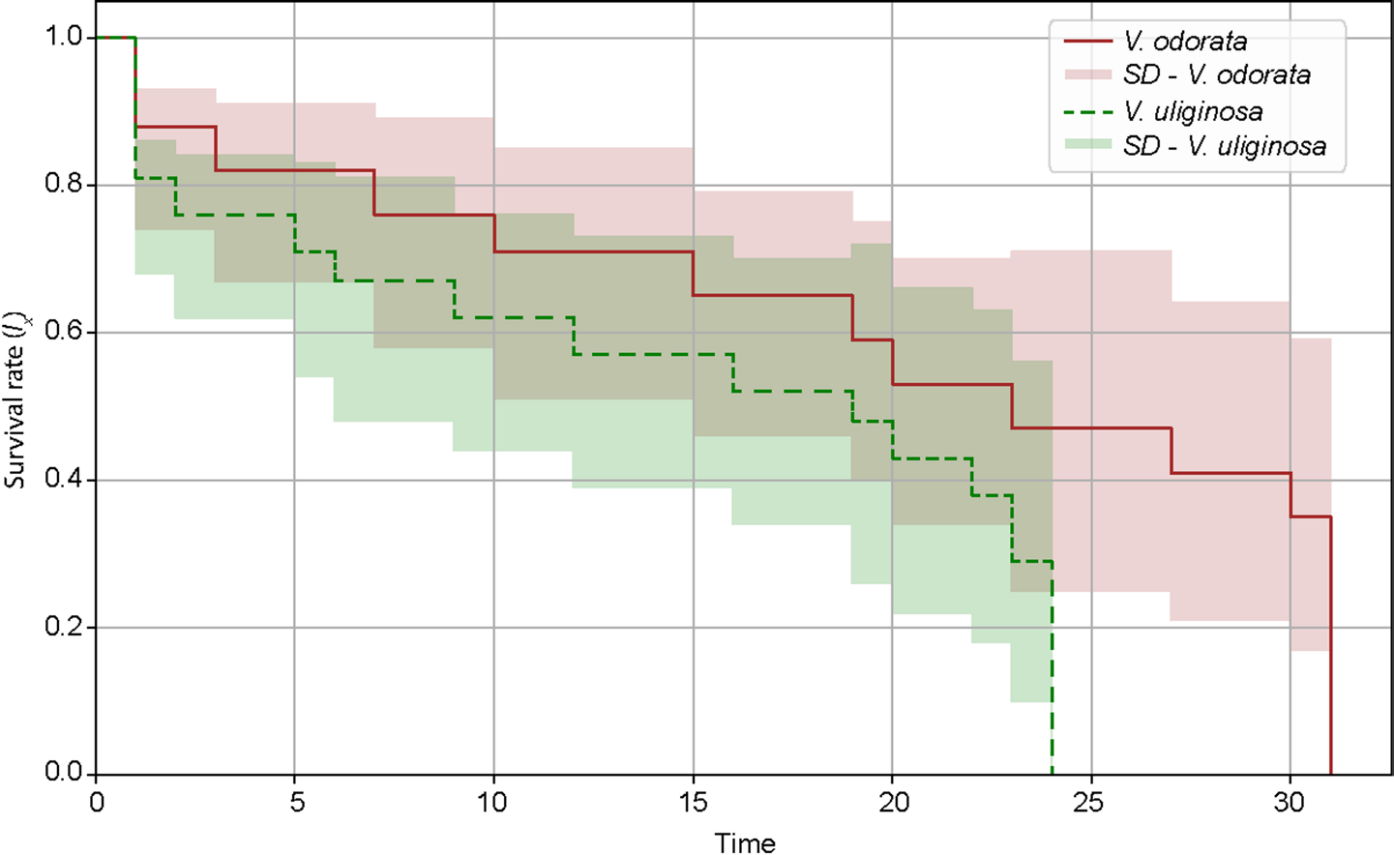
Survival curves of *Leipothrix violarius* females reared on *Viola odorata* (solid red line) and *Viola uliginosa* (dashed green line). The plot illustrates the proportion of surviving females over time, highlighting higher longevity and survivorship on *V. odorata* compared to *V. uliginosa*

The analysis of the survival rate of females revealed a clear difference in longevity between females bred on the two studied host plants (Fig. 1). On *V. odorata*, females exhibited a higher survival rate throughout the observation period compared to females reared on *V. uliginosa*. The survival curve for *V. odorata* (solid red line) remained above that of *V. uliginosa* (dashed green line), which indicates a greater overall survivorship.

**Fig. 2:**
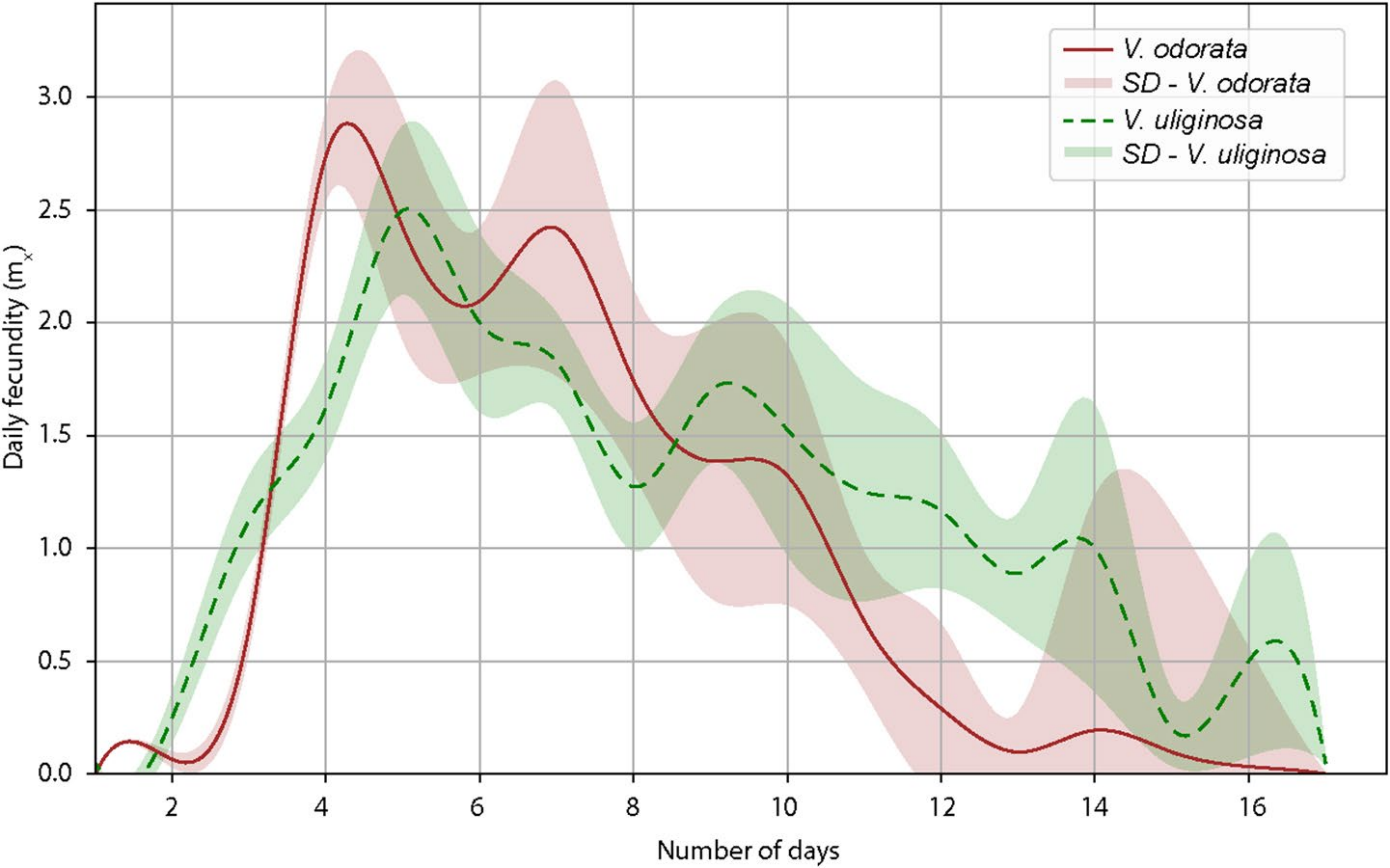
Daily fecundity of *Leipothrix violarius* females reared on *Viola odorata* (solid red line) and *Viola uliginosa* (dashed green line). The graph shows the mean number of eggs laid per female per day over a 14-day period

Female reproductive parameters did not show significant differences between the two populations reared on different host plants (Table 3). Females on *V. odorata* exhibited a rapid increase in daily fecundity, reaching their peak (approximately 3.0 offspring per day) on the 5th day, followed by a sharp decline after the 10th day (Fig. 2). In contrast, on *V. uliginosa*, females reached their peak (~ 2.5 offspring per day) slightly later, around day 6th, and maintained relatively stable fecundity levels from day 7 to day 14. Statistical analysis revealed no significant variation in sex ratio between plant species (Table 3).

**Table 3:** Reproductive parameters of *Leipothrix violarius* and females’ longevity bred on *Viola odorata* and *Viola uliginosa* (mean ± SD)

Parameters	*V. odorata*	*V. uliginosa*	*p*-value
Pre-ovipostion	2.45 ± 0.68	2.17 ± 0.82	0.162
Oviposition	6.13 ± 3.20	7.17 ± 3.13	0.234
Post-oviposition	1.45 ± 1.75	1.46 ± 1.82	0.989
Longevity of females	10.03 ± 2.85	11.71 ± 3.25	0.047
Total fecundity	16.23 ± 12.05	15.04 ± 8.20	0.681
Daily fecundity	1.45 ± 0.84	1.26 ± 0.61	0.355
n	31	24	
Sex ratio	0.74 ± 0.08	0.81 ± 0.08	0.540

### Life-table’s parameters

The life-table was constructed based on the above-provided data and was significantly better for mites reared on *V. odorata*. The average time of generation development of *L. violarius* on both plant species took about two weeks (Table 4). However, during this period, the cohorts reared on *V. odorata* increased by 30% more than on *V. uliginosa*. The doubling time for the population reared on *V. odorata* was almost one day shorter than that on *V. uliginosa*.

**Table 4:** Demographic parameters of *Leipothrix violarius* reared on *Viola odorata* and *Viola uliginosa* (mean ± SD)

Parameters	*V. odorata*	*V. uliginosa*	*p*-value
Reproductive rate (*R*_*0*_)	8.44 ± 1.01	6.24 ± 0.82	0.001
Mean generation time (*T*)	13.17 ± 0.34	13.79 ± 0.40	0.003
Intrinsic rate of population increase (*r*_*m*_)	0.16 ± 0.01	0.13 ± 0.01	< 0.001
Finite rate of population (λ)	1.18 ± 0.01	1.14 ± 0.01	< 0.001
Doubling Time (days) *TD*	4.30 ± 0.18	5.27 ± 0.35	< 0.001
n	31	24	

### Range of host plants

**Fig. 3:**
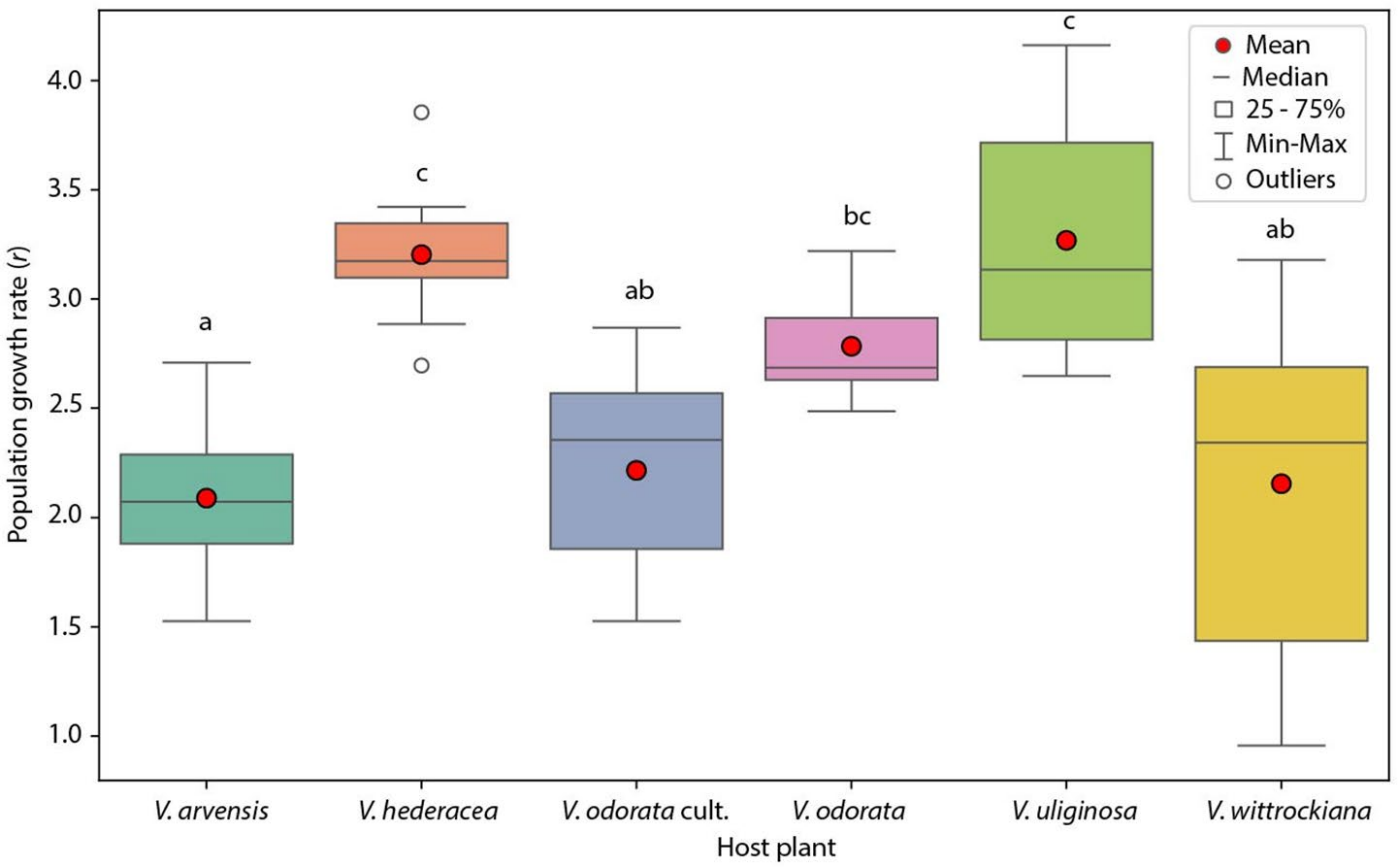
The population growth rate of *Leipothrix violarius* on different host plants. Min: lowest data point (excluding outliers), Q1: first quartile (25th percentile), median: middle value of the data (50th percentile), Q3: third quartile (75th percentile), max: highest data point (excluding outliers), mean: average value (red dot). The same letters represent homogeneous groups

The population growth rate was calculated to determine the host range of *L. violarius*. The value of this parameter showed the ability of the *L. violarius* population, adapted to *V. uliginosa*, to develop on all studied host species (Fig. 3). However, significant differences among populations developed on different host plants were noted (*p* = 0.0000). The highest growth rates were observed on *V. uliginosa* and *V. hederacea*, with no significant difference between these two violet species. Additionally, population reared on wild-collected *V. odorata* did not differ significantly from those reared on *V. uliginosa* and *V. hederacea*, nor from populations reared on *V. odorata* cult. and *V. wittrockiana*. In contrast, the lowest growth rate was observed on *V. arvensis*.

## Discussion

Our study demonstrated that all developmental, reproductive, and population parameters of *L. violarius* reared on *V. odorata* were similar to or in some cases better than those recorded on *V. uliginosa*. The latter plant was the source of the initial *L. violarius* population and is classified as endangered in parts of its range. Our data suggests no detectable adaptation cost when shifting from *V. uliginosa* to *V. odorata*. A comparable phenomenon was observed by Sybilska et al. (2025), who found no significant differences in the life-history traits of tomato russet mite (*A. lycopersici*) reared on tomato (*Solanum lycopersicum* L.) and black nightshade (*Solanum nigrum* L.). In that case, no cost of host switching was detected.

Using *L. violarius* as a model species to study cyclotide-mediated defences in epidermal feeders requires prior knowledge of its biology. This study provides a complete data set on its developmental, reproductive, and life-table parameters. Immatures completed their development in approximately seven days on both host plants—similar to the developmental time reported for *P. adalius* on detached rose leaves under comparable laboratory conditions (Druciarek et al. 2014), as well as *A. lycopersici* (Abou-Awad 1979; Haque and Kawai 2003) and *Metaculus mangiferae* (Attiah) (Abou-Awad et al. 2011). Other species require more time, such as *Retracarus johnstoni* Keifer, up to 20.5 days (Gondim and Moraes 2003) or *Phyllocoptes fructiphilus* Keifer, a known vector of *Rose rosette virus*, 11 days at 23 °C (Kassar and Amrine 1990). Despite similar longevity, *P. adalius* females laid nearly twice as many eggs as *L. violarius* females. The latter also had lower total and daily fecundity than *Leipothrix dipsavivagus* (Stoeva et al. 2011). Moreover, *A. lycopersici* females showed three times higher fecundity when reared under similar conditions (Haque and Kawai 2003).

*Leipothrix violarius* can be classified as a species with moderately high life-history parameters. Its intrinsic rate of natural increase (*r*_*m*_) on *V. odorata* was 0.16 day⁻¹, a parameter considered by Birch (1948) as one of the best indicators of population adaptability. This value is comparable to other vagrant eriophyoids: 0.15 for *Aculus fockeui* (Nalepa & Trouessart) (Abou-Awad et al. 2010), 0.14 for *Calepitrimerus vitis* (Nalepa) (Walton et al. 2010), 0.11 for *Aceria mangiferae* Sayed and 0.14 for *M. mangiferae* (Abou-Awad et al. 2011), and 0.10 for *Phyllocoptruta oleivora* (Ashmead) (Allen et al. 1995). Slightly higher values were recorded for *P. adalius* 0.21 (Druciarek et al. 2014), and much higher for *A. lycopersici* 0.24–0.29 (Haque and Kawai 2003; Xu Xiang et al. 2006). The moderately high *r*_*m*_ observed in our study suggests that *L. violarius* can be effectively reared in laboratory conditions, enabling the creation of stable stock populations for future experiments.

Besides *V. odorata*, other *Viola* species can also serve as suitable hosts for *L. violarius*, as favourable population growth rates were recorded for all tested species. The lowest value was observed on *V. arvensis*. All tested *Viola* species—except *V. × wittrockiana*—have been previously analyzed for their cyclotide profiles, with the number of identified compounds ranging from 20 in *V. uliginosa* to 66 in *V. hederacea* (Burman et al. 2015; Craik et al. 1999; Göransson et al. 2004, 2004; Slazak et al. 2016; Trabi and Craik 2004). The variation in population growth rates across tested *Viola* species may be partially explained by their phylogenetic relationships, which could influence plant traits such as secondary metabolite profiles and tissue suitability for eriophyoid feeding (Agrawal and Fishbein 2006; Wink 2003). Additionally, we cannot exclude the possibility that the variation may be attributed to the cyclotide composition in different *Viola* species. Cyclotides have been positively identified in at least 150 species of Violaceae family, each containing a unique set of 1 to 25 compounds (Burman et al. 2015; Daly et al. 2009; Weidmann and Craik 2016). These biologically active peptides are naturally synthesized by plants, exceptionally stable in structure, and are well known for their bioactive potential (Daly et al. 2009; Weidmann and Craik 2016). Their antifungal, nematocidal, molluscicidal, and insecticidal properties strongly support their role as plant defence compounds, with confirmed activity against a broad range of organisms including nematodes (Colgrave et al. 2008; Gilding et al. 2016), trematodes (Malagón et al. 2013), molluscs (Plan et al. 2008) and various insects (Jennings et al. 2001, 2005; Pinto et al. 2012).

It is important to note that cyclotides are not evenly distributed or stored across different plant organs or tissues. Their distribution appears functionally linked to biological roles, showing variation across seasons and environmental conditions (Simonsen et al. 2005; Slazak et al. 2016; Trabi et al. 2004). Slazak et al. (2022) demonstrated that the composition of cyclotides in *V. odorata* leaves can be modulated by herbivory, as higher levels of specific peptides were detected in tissues infested by the two-spotted spider mite compared to non-infested controls. This finding suggests that cyclotide expression is inducible in response to mite feeding. Although this phenomenon has not yet been examined in the context of eriophyoid mites, the confirmed presence of cyclotides in epidermal cells indicates that a similar response may occur. Therefore, studies analogous to (Slazak et al. 2022) are needed to test whether epidermis-feeding eriophyoids elicit comparable changes. We propose that *L. violarius* and *V. odorata* provide a promising model system for investigating these interactions.

As shown by Slazak et al. (2016), not only the number but also the spatial distribution of cyclotides differs between *Viola* species: in *V. uliginosa*, cyclotides are evenly distributed in the petiole epidermis, whereas in *V. odorata*, they are particularly abundant in the leaf epidermis. These differences in tissue-specific cyclotide accumulation may partly explain the variation in mite performance observed across host species, especially considering that *L. violarius* feeds primarily on epidermal cells of the leaves. The shorter lifespan of *L. violarius* females on *V. odorata*, despite their similar fecundity compared to those reared on *V. uliginosa*, may suggest reduced food quality, potentially linked to cyclotides’ presence or specific composition. This difference may also contribute to the variation observed in egg development time, which was shorter on *V. odorata*. Since the egg stage is non-feeding, such differences likely result from bioactive compounds ingested by the female prior to oviposition. Cyclotides have been shown to pass through the digestive systems of herbivores and affect their internal physiology. This was demonstrated by Dancewicz et al. (2020), who found that aphids ingesting cyclotides via phloem sap exhibited impaired midgut function and reduced feeding acceptance. Similarly, Slazak et al. (2022) reported that *Tetranychus urticae* Koch feeding on cyclotide-rich mesophyll tissues transported these peptides to their ovaries and eggs, negatively affecting reproduction. Our findings suggest that cyclotides ingested by *L. violarius* may similarly influence its survival or reproductive performance.

Given its strict host specificity and exclusive epidermal feeding behaviour, *L. violarius* emerges as a promising model species for studying the complex interactions between cyclotide-producing plants and herbivorous arthropods that feed on the outermost plant cell layers. Unlike other well-studied arthropods such as *Myzus persicae* Sulzer or *T. urticae*, which target phloem or mesophyll, *L. violarius* specializes in epidermal tissues—where cyclotides have been directly detected (Slazak et al. 2022). This feeding habit, combined with the mite’s ability to colonize multiple *Viola* species differing in both the number and spatial distribution of cyclotides, makes it uniquely suited for disentangling the effects of these peptides on epidermis-feeding organisms. Furthermore, the fact that *L. violarius* can complete development and maintain reproductive success on hosts with distinct cyclotide profiles—such as *V. odorata* and *V. uliginosa*—suggests that it may have evolved specific physiological mechanisms to tolerate, circumvent, or possibly manipulate these plant defences. Such adaptations are largely unknown in eriophyoids and warrant detailed physiological and molecular investigation. At the same time, this system offers an excellent opportunity to test whether *L. violarius* feeding induces differential expression of cyclotides in plant epidermis, as has been shown for other herbivores feeding on deeper tissues (Slazak et al. 2022). With standardized rearing protocols now established and complete life-table data available, *L. violarius* can be used in controlled experimental setups, e.g., gene expression assays or peptide profiling—to test the bidirectional dynamics between plant cyclotide response and mite adaptation.

In summary, *L. violarius* represents a rare example of an epidermis-feeding eriophyoid that can be reliably maintained under laboratory conditions and manipulated across different *Viola* species. This makes it an exceptionally valuable model for exploring (1) how epidermal herbivory influences cyclotide biosynthesis and distribution, and (2) how these defence peptides affect the biology of mites with extreme feeding specialization. Such studies may not only clarify the ecological role of cyclotides in epidermal defence but also shed light on the co-evolutionary trajectories between *Viola* plants and their highly adapted herbivores.

## Data Availability

The datasets generated during and/or analysed during the current study are available from the corresponding author on reasonable request.
